# The role of blood related inflammatory factors on age-related macular degeneration (AMD)

**DOI:** 10.1186/s12979-024-00440-5

**Published:** 2024-06-05

**Authors:** Habib Ojaghi, Shirin Poorsheykhian, Amin Najafi, Sohrab Iranpour

**Affiliations:** 1 Department of Surgery, Ardabil University of Medical Sciences, Ardabil, Iran; 2 MD, Ardabil University of Medical Sciences, Ardabil, Iran; 3 Department of Epidemiology, Ardabil University of Medical Sciences, Ardabil, Iran

**Keywords:** Lymphocyte, Monocyte, Neutrophil, C-reactive protein, Age-related macular degeneration

## Abstract

**Background:**

Age-related macular degeneration (AMD) is a significant retinal disease that leads to irreversible low vision, particularly in developing countries. The variation in AMD prevalence among different racial groups and highlighted role of inflammation on disease pathology from previous studies which yielded in inconsistent findings, It seems to be of great importance to do more investigation in this field.

**Methods:**

This case control study involved 204 participants, divided into four groups of equal size (51 individuals per group). Three groups represented AMD cases of varying severity according to Beckman classification (3 groups) and one healthy control group. Sampling was conducted exhaustively until the desired sample size was reached. The control group comprised healthy individuals without any infectious or inflammatory systemic, ophthalmic disease. Blood samples were collected to measure inflammatory factors, including lymphocytes, monocytes, neutrophils, neutrophil-to-lymphocyte ratio (NLR), and C-reactive protein (CRP). Collected data were analyzed by statistical methods in SPSS version 21.

**Results:**

Of the participants, 51% were women, and their ages ranged from 47 to 89 years (62.2 ± 8). According to multiple logistic regression analysis, age exhibited a statistically significant positive association with AMD severity (*P* = 0.038, odds ratio [OR] = 1.034). ANOVA results indicated a significant association between neutrophil count and AMD severity (*P* < 0.001). As the disease severity increased, the number of neutrophils decreased. The mean ± SD neutrophil counts for early, intermediate and advanced AMD were 3849 ± 800, 3702 ± 734, and 3342 ± 823, respectively. No statistically significant associations were found between lymphocyte count, monocyte count, neutrophil-to-lymphocyte ratio, CRP, and AMD.

**Conclusion:**

There was a significant relationship between the number of neutrophils in peripheral blood and the severity of AMD in study participants which needs more evaluation for the potential utility of this factor in the prognosis of AMD. There was not any significant relationship among the other factors and AMD.

## Introduction

The retina is a highly intricate tissue with a well-defined structure which plays a crucial role in capturing visual images and converting them into electrical signals to be transferred through the optic nerve to the visual cortex for processing. These signals contribute to our perception of various visual aspects such as form, color, contrast, spatial positioning, depth, and movement [1].

Age-related macular degeneration (AMD) is a significant retinal disease and a leading cause of irreversible vision loss, particularly in developing countries [2]. Elderly individuals are particularly vulnerable to AMD, which is considered the primary cause of permanent blindness in developed nations [3]. In advanced stages, AMD severely impairs the ability to carry out daily activities, including reading, facial recognition, and driving. The global prevalence of AMD is estimated to affect approximately 170 million people, with around 8.7% of individuals over 45 years old being affected. There are manifested cases of AMD under 40 years old as well. AMD stands in the first rank among the causes of visual impairments in industrialized countries and third one worldwide. Given the aging population, the prevalence of AMD is projected to reach 288 million by 2040, leading to escalating treatment and care costs for affected individuals [4–6].

AMD is a neurodegenerative disease primarily targeting the macular region of the retina. While the exact cause is not fully understood, pathological findings include yellow subretinal deposits, alterations in the retinal pigment epithelium (RPE), geographic atrophy, and choroidal neovascularization (CNV) [7]. During the disease progression, abnormal blood vessels grow through the beneath of Bruch’s layer and subsequently spread to the sub retinal area. This process results in subretinal hemorrhages, serous retinal detachment, disciform scars, and retinal atrophy. The early stages of AMD are characterized by drusen presence, while advanced stages exhibit atrophy of the retinal pigment epithelium and the spread of choroidal new vessel through Bruch’s layer into the retina [8]. This occurrence leads to exudative or hemorrhagic changes.

AMD is classified into dry and wet types. Wet AMD, also known as neovascular AMD, is associated with exudative manifestations, whereas dry AMD, including primary AMD and geographic AMD, presents with non-exudative clinical features [9]. Some studies have suggested that antioxidants and carotenoid compounds may have a protective effect against AMD, highlighting the importance of factors such as diet and a healthy lifestyle [10–12].

Inflammation seems to be an important factor in AMD pathology. The presence of inflammatory factors in drusens and recent established role of complement cascade on disease are in favor of this theory. The count of the blood WBC and their ratio together is considered to be as a factor for evaluation of the body inflammatory condition. Neutrophil to Lymphocyte Ratio (NLR) recently introduced as an inflammatory factor that mostly represent the balance between innate and acquired immunity. The NLR recently is supposed a potential factor in progression of AMD [4–7].

This study aimed to explore the relationship between peripheral blood inflammatory factors and AMD disease.

## Materials and methods

A case control analytical study was conducted on patients diagnosed with AMD, who were referred to the eye clinic of Imam Reza hospital in Ardabil, from January 2022 to August 2023. The case group included of individuals meeting the AMD criteria. Exclusion criteria included the presence of any infectious or inflammatory systemic diseases or any other ophthalmic disease especially retinal disease. Smokers also excludes from the study because of the confounding effect of smoking on disease.

Sample Size: The total sample size using G Power software (error level of 5%, power of 85%, effect size of 25%) was 204 cases in total which equally divided into 4 groups with each group containing of 51 individuals.

**Fig. 1:**
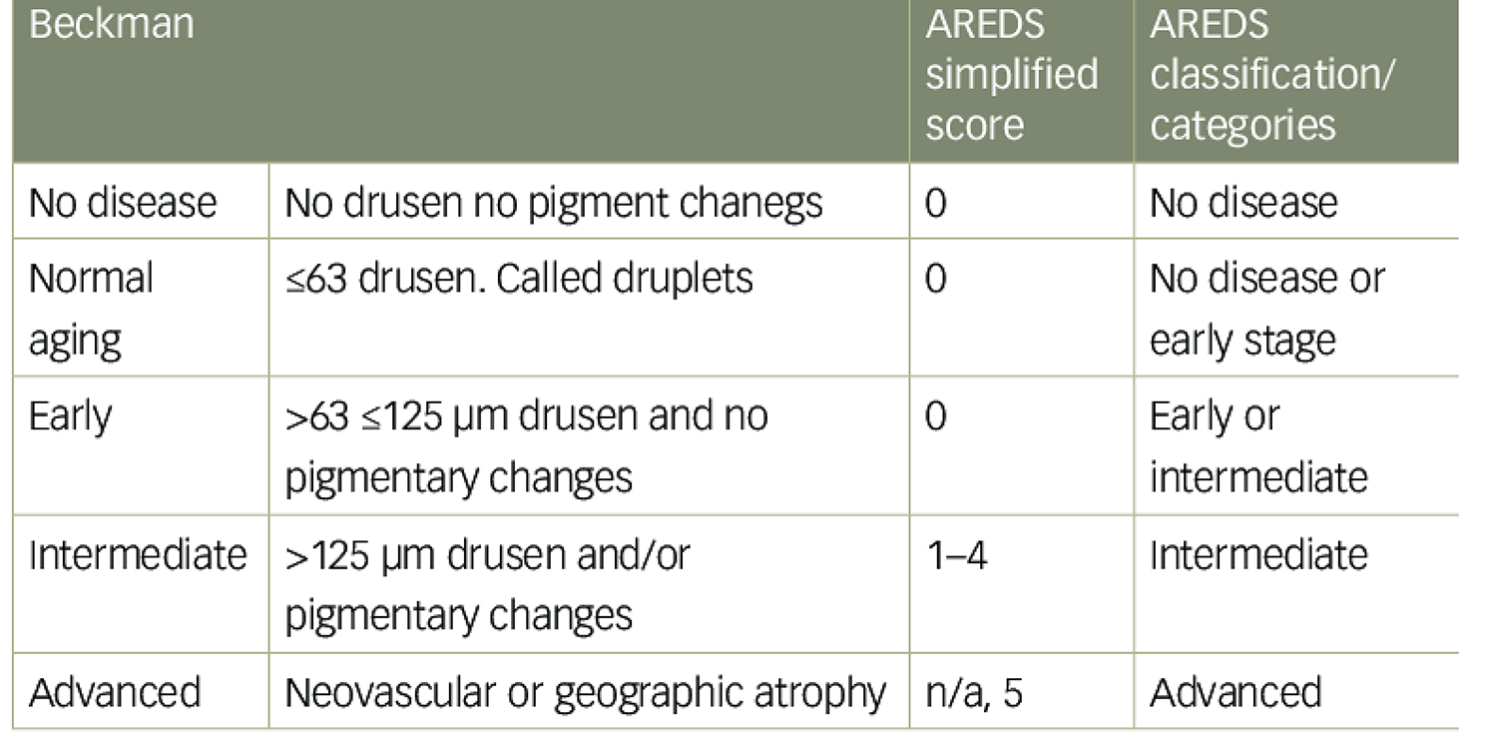
Beck man classification for AMD

Procedure: All patients underwent a thorough ocular examination including best-corrected visual acuity measurement (AnnoTek LED Vision Chart; LC-1300B), anterior segment examination using a slit-lamp (Topcon SL-3 C), IOP measurement using Goldmann’s applanation tonometry (Haag Streit-AT900), and fundus examination under a slit-lamp using a Volk 90D lens (Volk Optical Inc.) and using a binocular indirect ophthalmoscope (HEINE OMEGA-100) using a Volk 20D lens (Volk Optical Inc.). Individuals diagnosed with AMD were classified into one of three stages: early, intermediate, or advanced according to Beckman classification criteria (Fig. 1). Visual acuity was also measured. Blood samples were collected from the participants to test inflammatory factors (lymphocytes, monocytes, neutrophils, neutrophil-to-lymphocyte ratio, and CRP) in the center’s laboratory. The time between sampling and analysis was less than 1 h. Patient information, including age, sex, and levels of inflammatory factors, was recorded in the study checklist.

Qualitative Bionic kit used for CRP analysis which reports the results as negative, 1+, 2 + and 3+.

In the case group, patients were categorized into three subgroups based on examination findings for disease classification. A healthy control group with no ocular or systemic disease was also included (the same ocular examination done for control group).

Data Analysis: The collected data was analyzed using SPSS version 21 software. Analysis of variance (ANOVA), Fisher’s exact test, and ordinal logistic regression were used to investigate the relationship between inflammatory factors and AMD disease while controlling for confounding variables. A significance level of *P* < 0.5 was considered statistically significant.

Ethical Considerations: All participants were assured of the confidentiality of their information and that their involvement in the study was solely for research purposes. Informed consent was obtained from all participants, and they answered the research questions with satisfaction. This research received ethical approval from the ethics committee of Islamic Azad University, Ardabil branch, with the code of ethics IR.ARUMS.MEDICIN.REC.1401.080.

## Results

This study included a total of 204 participants, the age distribution of the patients in the study ranged from 47 to 89 years, with a mean age of 62.2 ± 8 years. According to the ANOVA test, there was no statistically significant relationship between the age variable and the severity of AMD disease (*P* = 0.052), (Table 1).

**Table 1:** Age distribution of study participants in the Patient group (AMD) and control

*P*-Value^*^	Age			Number	Groups under study	
	Maximum	Minimum	Mean ± SD			
0.052	89	48	62.1 ± 8	51	Control	
	80	48	60.5 ± 7	51	mild	AMD
	80	47	61.4 ± 8	51	intermediate	
	81	49	64.8 ± 7	51	advanced	
	89	47	62.2 ± 8	204	Total	

^*^ANOVA

The study included a total of 204 participants, of which 104 (51%) were women and 100 (49%) were men. According to the chi-square test, there was no statistically significant relationship between gender and the severity of AMD disease (*P* = 0.666). Among the studied groups, 56.9% of women and 43.1% of men had an advanced condition of AMD (Table 2).

**Table 2:** Frequency distribution of gender of study participants in patient (AMD) and control groups

*P*- Value^*^	Sex						Groups under study	
	Total		Male		Female			
	%	Number	%	Number	%	Number		
0.666	100	51	51	26	49	25	Control	
	100	51	47.1	24	52.9	27	mild	AMD
	100	51	54.9	28	45.1	23	intermediate	
	100	51	43.1	22	56.9	29	advanced	
	100	204	49	100	51	104	Total	

^*^Chi-Square

The analysis of lymphocyte counts showed that, although the mean and standard deviation of lymphocyte numbers were lower in the patient groups with AMD compared to the control group with healthy eyes, there was no statistically significant relationship between the number of lymphocytes and the severity of AMD disease (*P* = 0.139), (Table 3).

**Table 3:** Relationship between the number of peripheral blood lymphocytes and age-related macular degeneration disease

*P* Value^*^	Lymphocyte (10*3/micro litter)			Number of persons	Groups under study	
	Maximum	Minimum	Mean ± SD			
0.139	3200	1080	2001 ± 524	51	Control	
	2750	270	1902 ± 362	51	mild	AMD
	2800	1330	1811 ± 366	51	intermediate	
	2980	1320	1929 ± 371	51	advanced	
	3200	270	1911± 411	204	Total	

Regarding peripheral blood monocytes, there was no statistically significant relationship between the number of monocytes and the severity of AMD disease (*P* = 0.203). The mean and standard deviation of monocyte counts in participants with mild, intermediate, and advanced AMD were 604 ± 114, 573 ± 97, and 756 ± 909, respectively.

Table 4 presents the relationship between the number of neutrophils and the severity of AMD. The ANOVA test revealed a significant relationship (*P* < 0.001) between the number of neutrophils and AMD severity. The control group had the highest average number of neutrophils (4134), indicating that as the disease severity increased, and the number of neutrophils in peripheral blood decreased. The mean and standard deviation of neutrophil counts in mild, intermediate, and advanced AMD cases were 3849 ± 800, 3702 ± 734, and 3342 ± 823, respectively. Pairwise comparisons based on Tukey and LSD test showed that the number of neutrophils in the control group was significantly different from the moderate and severe AMD groups (*P* = 0.028 and *P* < 0.001, respectively), with average differences of 431.7 and 791.9. Additionally, there was a significant difference between mild and severe AMD (*P* = 0.006), with an average difference of 507.3.

**Table 4:** Relationship between the number of peripheral blood neutrophils and age-related macular degeneration

*P*-Value^*^	Neutrophil (10*3/micro litter)			Number	Groups under study	
	Maximum	Minimum	Mean ± SD			
P_1,2,3,4_<0.001 P_1,2_=0.255 P_1,3_=0.028 P_1,4_<0.001 P_2,3_=0.776 P_2,4_=0.006 P_3,4_=0.093	5720	3020	4134 ± 610	51	Control	
	5800	2450	3849 ± 800	51	mild	AMD
	5000	1520	3702 ± 734	51	intermediate	
	8790	2050	3342 ± 932	51	advanced	
	8790	1520	3757± 823	204	Total	

^*^ANOVA and Tukey LSD

The relationship between the ratio of neutrophils to peripheral blood lymphocytes (NLR) in the studied patients with age-related macular degeneration (AMD) was investigated. The results indicated a relationship between this ratio and the severity of AMD, as determined by the ANOVA test. However, no statistically significant correlation was observed in the studied patients (*P* = 0.178). The mean and standard deviation of this ratio in mild, intermediate, and advanced AMD cases were 2.40 ± 2.77, 2.10 ± 0.55, and 1.76 ± 90.43, respectively. As the severity of AMD increased, the ratio between the number of neutrophils and the number of lymphocytes in peripheral blood showed a further decrease.

**Fig. 2:**
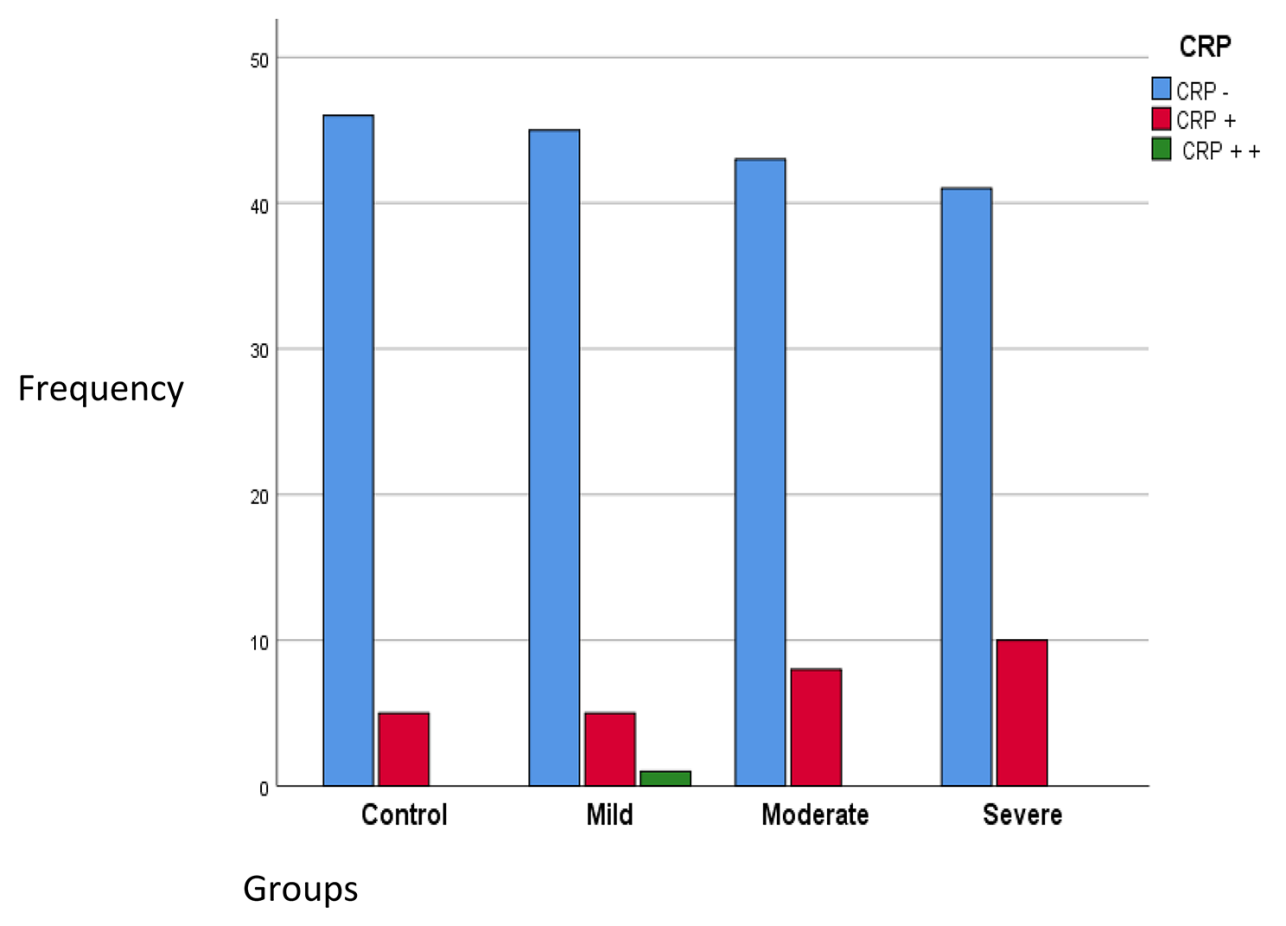
Frequency distribution of CRP status in the studied groups

The relationship between C-reactive protein (CRP) in peripheral blood and age-related macular degeneration was also examined. No significant relationship was found between different values of CRP (negative, 1 positive, and 2 positive) and the severity of AMD (*P* = 0.434). However, the presence of CRP + 1 increased in mild, intermediate, and advanced AMD conditions, observed in 9.8%, 15.7%, and 19.6% of the study participants, respectively. Although the percentage of CRP + increased with the severity of AMD, but this increase was not statistically significant (*P* = 0.439), (Fig. 2).

In the multivariate analysis using the ordinal logistic regression model, the relationship between blood inflammatory factors and the severity of AMD was examined based on the information presented in Table 5. Among the studied variables, age showed a positive relationship (*P* = 0.038 and OR = 1.034) with AMD disease, indicating that increasing age was associated with a higher likelihood of AMD. On the other hand, the number of neutrophils demonstrated a significant inverse relationship (*P* < 0.001 and OR = 0.999) with AMD, suggesting that as the severity of the disease increased, the number of neutrophils decreased. The other variables analyzed did not show a significant relationship with the severity of AMD.

**Table 5:** Multiple analysis based on ordinal logistic regression model

Estimated values						
95% confidence interval relative odds		Odd Ratio	*p*-value ^*^ (P)	standard error (St. Error)	regression coefficient (B)	Variable
Upper limit	lower limit					
1.573	0.562	0.941	0.815	0.2625	-0.061	Male
0	0	1	0	0	^a^.	Female
23.888	0.066	1.254	0.881	1.5038	0.226	CRP -
53.604	0.129	1.625	0.531	1.539	0.965	CRP 1+
0	0	1	0	0	^a^.	CRP 2+
1.067	1.032	1.034	0.038	0.0162	0.33	Age
1.1	0.999	1.0	0.391	0.0004	0	Lymphocyte
1.003	0.999	1.001	0.409	0.0011	0.001	Monocyte
0.999	0.899	0.999	0.0	0.0002	-0.001	Neutrophil
1.287	0.873	1.06	0.557	0.099	0.058	NLR^**^

^*^Ordinal Logistic Regression ^**^Neutrophil/Lymphocyte Ratio

## Discussion

Age-related macular degeneration (AMD) is a significant retinal disease and a leading cause of irreversible vision loss in developing countries. The variation in AMD prevalence among different races and the contradictory findings in related studies have underscored the importance of investigating this disease [13]. Consequently, this study aims to explore the association between inflammatory factors in peripheral blood and AMD severity.

The findings of this study indicate that there is no statistically significant relationship between the age of the patients and the severity of AMD (*P* = 0.052). However, the mean and standard deviation of mild, intermediate, and advanced AMD cases demonstrate an increase in the prevalence of severe disease status with the rise in the average age of the patients. These results align with prior articles and sources that have associated macular degeneration with aging. For instance, Heesterbeek, Pugazhendhi, and their colleagues conducted separate studies in the United States and the Netherlands, respectively, examining AMD risk factors [14, 15].

Their studies, published in 2020 and 2021, respectively, denoted AMD as a common progressive disease predominantly affecting the elderly, leading to progressive visual impairment. Similarly, Ilhan et al. reported in their study in Turkey that there is a direct correlation between age and the severity of AMD [8].

The Chi-Square test reveals no statistically significant relationship between the gender variable of the sampled individuals and the severity of AMD. However, the severe condition of AMD is observed in 56.9% of female participants compared to 43.1% of males. Therefore, the outcomes of this study do not reveal a significant association between age and AMD prevalence but confirm a higher prevalence of severe AMD in women compared to men. Previous studies have also suggested gender (female) as one of the risk factors for macular degeneration. Heesterbeek et al., in their article on AMD risk factors, mentioned gender as being loosely associated with the occurrence of the disease [14]. Other potential risk factors include family history, alcohol consumption, diabetes, high blood pressure, light iris color, high serum cholesterol level, cardiovascular disease, lens opacities, and previous cataract surgery [3, 7]. Environmental risk factors such as white race, female gender, and smoking are also considered in terms of AMD risk factors [1, 6]. In a study by Kurtul et al., gender was identified as a weak predictor of AMD, and no statistically significant relationship was reported (*P* = 0.058) [7].

In assessing the relationship between the number of peripheral blood lymphocytes and age-related macular degeneration, the data indicate that although the mean and standard deviation of lymphocyte count in the AMD study group were lower than the control group, no statistically significant correlation is observed between the number of peripheral blood lymphocytes and AMD severity in the studied patients. These findings differ from certain studies investigating changes in lymphocyte count in AMD patients. For example, a case-control study by Hector et al. in 2017 explored the association between B lymphocytes, monocytes, and AMD. The study reported no significant relationship between lymphocyte count in the control group and AMD disease (*P* = 0.603) [11].

In the study conducted by Litwinska et al. in Poland in 2019, entitled “Interaction between Systemic Inflammatory Factors in AMD,” a correlation was observed between the numbers of lymphocytes in AMD patients, whereas no correlation was found in the control group [16]. The difference in results between this study and the present study may be attributed to the sample size and other environmental factors that may have an influence on the study outcomes. Therefore, for more reliable conclusions, studies with larger sample sizes conducted at a broader scale, while controlling for confounding variables, are necessary.

Regarding the relationship between the number of peripheral blood monocytes and age-related macular degeneration, the data showed no statistically significant correlation between the number of monocytes and the severity of AMD. On average, individuals with mild and intermediate AMD conditions had lower numbers of monocytes compared to healthy individuals, whereas in advanced AMD cases, the average number of monocytes increased significantly. These findings align with the study conducted by Pinna et al. in 2018, which found that AMD patients had a lower total number of monocytes compared to the control group [17]. Similarly, a descriptive cross-sectional study by Xue et al. in 2021, focusing on the Chinese population, reported a positive relationship between the prevalence of AMD and higher levels of peripheral blood monocytes [18].

In analyzing the relationship between the number of peripheral blood neutrophils and age-related macular degeneration, a significant correlation was found between the number of neutrophils and the severity of AMD (*P* < 0.001). As the disease severity increased, the average number of neutrophils decreased. Pairwise comparisons of neutrophil numbers using the Tukey HSD test revealed a significant difference between the control group and the intermediate (*P* = 0.028) and advanced (*P* < 0.001) AMD groups. Furthermore, there was a significant difference in neutrophil numbers between mild and advanced AMD cases (*P* = 0.006). These results are consistent with the study conducted by Pinna et al. in Italy, which showed that AMD patients had a lower number of neutrophils compared to the control group [17].Beside that neutrophils are considered as the main arm of innate immunity system which gets weaker with aging, so decrease in neutrophils maybe a normal senile condition rather than a prognostic factor for AMD that needs more studies with larger sample size.

Regarding the ratio of neutrophils to lymphocytes and age-related macular degeneration, the data indicated a statistically significant relationship between the ratio and the severity of AMD in the study sample. As the severity of AMD increased, the average ratio between neutrophils and lymphocytes in the peripheral blood exhibited a greater decrease. These findings are in line with the study conducted by Pinna et al., which investigated the relationship between inflammatory biomarkers and AMD disease. The study included 158 participants divided into two equal groups: individuals with AMD and controls. The study determined that the ratio of neutrophils to lymphocytes was an unreliable biomarker in AMD patients [17]. However, in a study by Kurtul et al. in Turkey, aiming to explore the possible relationship between the neutrophil-to-lymphocyte ratio (NLR) and age-related macular degeneration, conducted on 120 participants, the study reported that NLR was associated with wet AMD as an independent factor [7].

Despite the cases mentioned above, the neutrophil-to-lymphocyte ratio (NLR) has been proposed as a marker of systemic inflammation in recent years. This ratio reflects the dynamic relationship between the innate immune system (neutrophils) and the acquired cellular immune system (lymphocytes). NLR serves as an indicator of the balance between systemic inflammation and the immune system and can be utilized as a prognostic factor in various diseases. Additionally, the impact of NLR in AMD patients has been examined in other studies [19, 20].

In assessing the relationship between C-reactive protein (CRP) and age-related macular degeneration in the study participants, the findings indicate that there was no significant correlation between different values of the CRP variable (negative, 1 positive, and 2 positive) and the severity of AMD. However, as the severity of the disease increased, a higher percentage of patients exhibited CRP + values. Consistent with the present study, Kurtul et al.‘s study, aiming to investigate the possible relationship between CRP and AMD, reported increased CRP levels in the AMD patient group compared to the control group [7]. Seddon et al. also reported in their study that high levels of CRP are independently associated with an increased risk of AMD, emphasizing the role of inflammation and genetic factors in AMD development [21].

## Conclusion

In summary, the present study demonstrates a direct relationship between the increasing severity of AMD and the higher average age of participants, confirming the association between age and macular degeneration. Moreover, the statistically significant correlation between the severity of AMD and the descending number of neutrophils in the peripheral blood supports the potential role of this factor in the severity of AMD which is considered as a new theory and needs more evaluation to be proved.

## Data Availability

The datasets used and/or analyzed in the present study are available from the corresponding author upon reasonable request.
